# Supplementary material for: Deletion of wfs1 Impairs Oligodendrocyte Precursor Cells Dorsal Distribution and Myelination Through the wfs1-hmgcs1 Axis in Zebrafish
Source: Biology (Basel). 2026 Aug 16;15(16):1402. doi: 10.3390/biology15161402 (PMC13510151; doi:10.3390/biology15161402)
Supplement: Supplementary file 1 [file biology-15-01402-s001.zip › File S1-Transcriptome sequencing data and differentially expressed genes (DEGs) in wfs1-- zebrafish at 5 dpf/2.KEGG/wfs1KOvsWT/DOWN/plot/wfs1KOvsWT.html]

### wfs1KOvsWT KEGG通路富集列表

---

```
## Warning: package 'knitr' was built under R version 3.5.1
```

| KEGGID | Description | GeneRatio | BgRatio | pvalue | padj | geneID | geneName | keggID | Count |
| --- | --- | --- | --- | --- | --- | --- | --- | --- | --- |
| dre00053 | Ascorbate and aldarate metabolism | 9/389 | 66/8379 | 0.0032293 | 0.4247597 | 100005587\_1/100536795/403070/406731/100415796/570913\_1/445326/767756/100006238\_1 | aldh9a1a.1/wu:fb14d09/rgn/ugt1ab/ugt5b5/ugt5g1/akr1a1a/ugt5a2/aldh9a1a.2 | dre:100005587/dre:100536795/dre:403070/dre:406731/dre:678597/dre:570913/dre:445326/dre:767756/dre:100006238 | 9 |
| dre00590 | Arachidonic acid metabolism | 9/389 | 72/8379 | 0.0058186 | 0.4247597 | 406449/767759/406370\_1/494108\_1/797309/566996\_1/399485/767688/100148818 | ptges3a/zgc:153704/pla2g6/ptgr1/cyp2j20/si:dkey-11k2.7/cyp2p10/zgc:154040/si:ch73-55i23.1 | dre:406449/dre:767759/dre:406370/dre:494108/dre:797309/dre:566996/dre:399485/dre:767688/dre:100148818 | 9 |
| dre04150 | mTOR signaling pathway | 20/389 | 251/8379 | 0.0127416 | 0.6108029 | 324140/336619/436718\_1/100333504\_1/359840\_1/100000252\_1/573492/792689\_1/79380\_1/245702\_1/563708/556087\_1/555584/58072/30369\_1/791993/790938/30127\_1/797250/497384\_1 | sgk1/eif4bb/rhocb/lamtor5/atp6v1b2/pik3r3b/rragca/mlst8/eif4ea/igf1rb/skp2/wnt2bb/prr5a/fzd8b/fzd7a/wnt4b/zgc:158423/wnt2/slc7a5/prkca | dre:324140/dre:336619/dre:436718/dre:100333504/dre:359840/dre:100000252/dre:573492/dre:792689/dre:79380/dre:245702/dre:563708/dre:556087/dre:555584/dre:58072/dre:30369/dre:791993/dre:790938/dre:30127/dre:797250/dre:497384 | 20 |
| dre00591 | Linoleic acid metabolism | 5/389 | 33/8379 | 0.0170594 | 0.6108029 | 406370\_1/797309/399485/767688/100148818 | pla2g6/cyp2j20/cyp2p10/zgc:154040/si:ch73-55i23.1 | dre:406370/dre:797309/dre:399485/dre:767688/dre:100148818 | 5 |
| dre00010 | Glycolysis / Gluconeogenesis | 10/389 | 103/8379 | 0.0209179 | 0.6108029 | 560753/100005587\_1/100536795/568001\_2/406339\_1/561416/393427/445326/436646/100006238\_1 | tpi1b/aldh9a1a.1/wu:fb14d09/pfkmb/hk2/pfkpb/zgc:63568/akr1a1a/galm/aldh9a1a.2 | dre:560753/dre:100005587/dre:100536795/dre:568001/dre:406339/dre:561416/dre:393427/dre:445326/dre:436646/dre:100006238 | 10 |
| dre01230 | Biosynthesis of amino acids | 10/389 | 109/8379 | 0.0296567 | 0.7216473 | 560753/568001\_2/322670\_1/550579/550524\_1/407673/561416/406688/334195/678634 | tpi1b/pfkmb/aco2/idh3g/cbsa/rpia/pfkpb/got2a/mat1a/mat2al | dre:560753/dre:568001/dre:322670/dre:550579/dre:550524/dre:407673/dre:561416/dre:406688/dre:334195/dre:678634 | 10 |
| dre00380 | Tryptophan metabolism | 7/389 | 67/8379 | 0.0349675 | 0.7293229 | 100005587\_1/100536795/368262/393860\_1/447801/571201/100006238\_1 | aldh9a1a.1/wu:fb14d09/dlst/gcdha/aldh8a1/si:ch73-97h19.2/aldh9a1a.2 | dre:100005587/dre:100536795/dre:368262/dre:393860/dre:447801/dre:571201/dre:100006238 | 7 |
| dre03410 | Base excision repair | 6/389 | 58/8379 | 0.0511537 | 0.7558405 | 321622/393774\_2/556995\_2/553405\_1/558198\_1/796446\_1 | hmgb1a/pole3/lig1/pole/si:ch211-141o9.10/adprhl2 | dre:110438871/dre:393774/dre:556995/dre:553405/dre:558198/dre:796446 | 6 |
| dre00270 | Cysteine and methionine metabolism | 7/389 | 73/8379 | 0.0518971 | 0.7558405 | 550524\_1/571201/791167/431773/406688/334195/678634 | cbsa/si:ch73-97h19.2/zgc:158222/enoph1/got2a/mat1a/mat2al | dre:550524/dre:571201/dre:791167/dre:431773/dre:406688/dre:334195/dre:678634 | 7 |
| dre00860 | Porphyrin metabolism | 7/389 | 74/8379 | 0.0551436 | 0.7558405 | 100002875\_1/406731/100415796/570913\_1/100329531\_1/64607\_1/767756 | hmox2a/ugt1ab/ugt5b5/ugt5g1/hmox2b/alas2/ugt5a2 | dre:100002875/dre:406731/dre:678597/dre:570913/dre:100329531/dre:64607/dre:767756 | 7 |
| dre01200 | Carbon metabolism | 13/389 | 173/8379 | 0.0587856 | 0.7558405 | 560753/568001\_2/322670\_1/550579/368262/407673/541329\_1/403070/792824\_1/406339\_1/561416/406688/393427 | tpi1b/pfkmb/aco2/idh3g/dlst/rpia/gcsha/rgn/pgp/hk2/pfkpb/got2a/zgc:63568 | dre:560753/dre:568001/dre:322670/dre:550579/dre:368262/dre:407673/dre:541329/dre:403070/dre:792824/dre:406339/dre:561416/dre:406688/dre:393427 | 13 |
| dre00983 | Drug metabolism - other enzymes | 10/389 | 124/8379 | 0.0621239 | 0.7558405 | 492517\_1/406731/100005763\_1/437016\_1/794381/100415796/570913\_1/406703/103910413/767756 | zgc:103586/ugt1ab/uck2a/tk2/si:dkey-78a14.4/ugt5b5/ugt5g1/gsta.1/tymp/ugt5a2 | dre:492517/dre:406731/dre:100005763/dre:437016/dre:794381/dre:678597/dre:570913/dre:406703/dre:103910413/dre:767756 | 10 |
| dre04810 | Regulation of actin cytoskeleton | 25/389 | 404/8379 | 0.0858626 | 0.7594997 | 57934/286739\_1/30429\_2/793158/436718\_1/566764/100000252\_1/557935/386856\_1/799354\_1/100005049/novel.946/100331581\_1/569566/571431/560564\_2/100536705\_2/553493/323285\_1/378480/393611/445305\_1/767646\_1/797491\_1/792699 | actb1/msna/mylpfa/cdc42l/rhocb/scin/pik3r3b/zmp:0000001082/pdgfra/cxcl12b/fgf1b/-/si:dkey-44g17.6/LOC569566/LOC571431/pdgfc/itgae.2/arhgef7a/scinla/pdgfaa/c6/arpc4l/abi2a/itgb7/rac1l | dre:57934/dre:286739/dre:30429/dre:793158/dre:436718/dre:566764/dre:100000252/dre:557935/dre:386856/dre:799354/dre:100005049/dre:386856/dre:100331581/dre:569566/dre:571431/dre:560564/dre:100536705/dre:553493/dre:323285/dre:103909601/dre:393611/dre:445305/dre:767646/dre:797491/dre:792699 | 25 |
| dre03015 | mRNA surveillance pathway | 9/389 | 118/8379 | 0.0972389 | 0.7594997 | 386630/324267/393693/100005893\_1/492341\_1/394058\_1/563172/562282\_1/559392 | tardbpl/hbs1l/sap18/magoh/pcf11/fus/etf1a/ppp2r5b/ppp2r2cb | dre:386630/dre:324267/dre:393693/dre:100005893/dre:492341/dre:394058/dre:563172/dre:562282/dre:559392 | 9 |
| dre00980 | Metabolism of xenobiotics by cytochrome P450 | 7/389 | 85/8379 | 0.0990888 | 0.7594997 | 406731/393293/100415796/570913\_1/406703/393427/767756 | ugt1ab/hsd11b1la/ugt5b5/ugt5g1/gsta.1/zgc:63568/ugt5a2 | dre:406731/dre:100006793/dre:678597/dre:570913/dre:406703/dre:393427/dre:767756 | 7 |
| dre00051 | Fructose and mannose metabolism | 5/389 | 54/8379 | 0.1042788 | 0.7594997 | 560753/568001\_2/406339\_1/561416/100007686 | tpi1b/pfkmb/hk2/pfkpb/LOC100007686 | dre:560753/dre:568001/dre:406339/dre:561416/dre:100007686 | 5 |
| dre00920 | Sulfur metabolism | 2/389 | 12/8379 | 0.1043725 | 0.7594997 | 777719/566675 | papss2a/suox | dre:777719/dre:566675 | 2 |
| dre00260 | Glycine, serine and threonine metabolism | 5/389 | 56/8379 | 0.1171012 | 0.7594997 | 394103\_1/541429\_1/550524\_1/541329\_1/64607\_1 | sardh/aoc2/cbsa/gcsha/alas2 | dre:394103/dre:541429/dre:550524/dre:541329/dre:64607 | 5 |
| dre00592 | alpha-Linolenic acid metabolism | 3/389 | 26/8379 | 0.1175570 | 0.7594997 | 406370\_1/767688/100148818 | pla2g6/zgc:154040/si:ch73-55i23.1 | dre:406370/dre:767688/dre:100148818 | 3 |
| dre00900 | Terpenoid backbone biosynthesis | 3/389 | 26/8379 | 0.1175570 | 0.7594997 | 334380/559054/100003383\_1 | zmpste24/hmgcra/pmvk | dre:334380/dre:559054/dre:100003383 | 3 |
| dre00410 | beta-Alanine metabolism | 4/389 | 41/8379 | 0.1208644 | 0.7594997 | 100005587\_1/541429\_1/100536795/100006238\_1 | aldh9a1a.1/aoc2/wu:fb14d09/aldh9a1a.2 | dre:100005587/dre:541429/dre:100536795/dre:100006238 | 4 |
| dre04217 | Necroptosis | 14/389 | 215/8379 | 0.1260831 | 0.7594997 | 336426\_1/559768/321622/373107\_1/337413/550542\_1/558816/100126128/564295\_2/368481/100148818/100149436\_1/561021\_1/403131 | chmp1b/zgc:173593/hmgb1a/birc2/stat6/chmp4c/LOC558816/zgc:173594/traf2a/stat1b/si:ch73-55i23.1/si:ch211-189a15.5/si:dkey-103e21.5/tlr4ba | dre:336426/dre:559768/dre:110438871/dre:373107/dre:337413/dre:550542/dre:558816/dre:100126128/dre:564295/dre:368481/dre:100148818/dre:100149436/dre:561021/dre:403131 | 14 |
| dre04120 | Ubiquitin mediated proteolysis | 13/389 | 199/8379 | 0.1344490 | 0.7594997 | 550134/564149/373107\_1/692325\_1/323951/323883/327215\_2/100136859/100001302/447935\_1/563708/393223\_1/30100 | ubb/pias2/birc2/flj11011l/klhl13/cul1a/cul5a/fbxw8/uba7/tceb1a/skp2/fzr1a/ube2kb | dre:550134/dre:564149/dre:373107/dre:692325/dre:323951/dre:323883/dre:327215/dre:100136859/dre:100001302/dre:447935/dre:563708/dre:393223/dre:30100 | 13 |
| dre00052 | Galactose metabolism | 4/389 | 43/8379 | 0.1373409 | 0.7594997 | 568001\_2/406339\_1/561416/436646 | pfkmb/hk2/pfkpb/galm | dre:568001/dre:406339/dre:561416/dre:436646 | 4 |
| dre00511 | Other glycan degradation | 3/389 | 28/8379 | 0.1386442 | 0.7594997 | 445250/445250\_1/100000684\_1 | neu3.2/neu3.2/neu3.5 | dre:445250/dre:445250/dre:100000684 | 3 |
| dre00030 | Pentose phosphate pathway | 4/389 | 45/8379 | 0.1546638 | 0.7594997 | 568001\_2/407673/403070/561416 | pfkmb/rpia/rgn/pfkpb | dre:568001/dre:407673/dre:403070/dre:561416 | 4 |
| dre04621 | NOD-like receptor signaling pathway | 13/389 | 205/8379 | 0.1573066 | 0.7594997 | 100000242/436718\_1/373107\_1/557239/436734\_1/564295\_2/323944/100334992/368481/100002946/561021\_1/403131/100149641 | zgc:92606/rhocb/birc2/antxr2a/txn/traf2a/si:ch1073-155h21.1/unm\_sa911/stat1b/cxcl8a/si:dkey-103e21.5/tlr4ba/LOC100149641 | dre:100000242/dre:436718/dre:373107/dre:557239/dre:436734/dre:564295/dre:323944/dre:100334992/dre:368481/dre:100002946/dre:561021/dre:403131/dre:100149641 | 13 |
| dre00040 | Pentose and glucuronate interconversions | 5/389 | 62/8379 | 0.1596665 | 0.7594997 | 406731/100415796/570913\_1/445326/767756 | ugt1ab/ugt5b5/ugt5g1/akr1a1a/ugt5a2 | dre:406731/dre:678597/dre:570913/dre:445326/dre:767756 | 5 |
| dre00340 | Histidine metabolism | 3/389 | 30/8379 | 0.1608932 | 0.7594997 | 100005587\_1/100536795/100006238\_1 | aldh9a1a.1/wu:fb14d09/aldh9a1a.2 | dre:100005587/dre:100536795/dre:100006238 | 3 |
| dre01210 | 2-Oxocarboxylic acid metabolism | 3/389 | 30/8379 | 0.1608932 | 0.7594997 | 322670\_1/550579/406688 | aco2/idh3g/got2a | dre:322670/dre:550579/dre:406688 | 3 |
| dre03030 | DNA replication | 4/389 | 48/8379 | 0.1820640 | 0.7594997 | 393774\_2/393241\_2/556995\_2/553405\_1 | pole3/rnaseh2b/lig1/pole | dre:393774/dre:393241/dre:556995/dre:553405 | 4 |
| dre00071 | Fatty acid degradation | 5/389 | 65/8379 | 0.1830034 | 0.7594997 | 100005587\_1/100536795/393860\_1/393427/100006238\_1 | aldh9a1a.1/wu:fb14d09/gcdha/zgc:63568/aldh9a1a.2 | dre:100005587/dre:100536795/dre:393860/dre:393427/dre:100006238 | 5 |
| dre04115 | p53 signaling pathway | 7/389 | 101/8379 | 0.1884674 | 0.7594997 | 140621/431763\_1/436699/140621\_1/796473\_1/100320152/561021\_1 | casp3a/gadd45ab/zgc:92658/casp3a/tp53i3/steap3/si:dkey-103e21.5 | dre:140621/dre:431763/dre:436699/dre:140621/dre:796473/dre:100320152/dre:561021 | 7 |
| dre04210 | Apoptosis | 14/389 | 232/8379 | 0.1903220 | 0.7594997 | 57934/140621/373107\_1/431763\_1/791531\_1/100000252\_1/552927\_1/564295\_2/140621\_1/58100\_1/559564/561021\_1/246726/558237\_1 | actb1/casp3a/birc2/gadd45ab/casp6l1/pik3r3b/casp6/traf2a/casp3a/badb/eif2ak3/si:dkey-103e21.5/birc5b/dab2ipb | dre:57934/dre:140621/dre:373107/dre:431763/dre:791531/dre:100000252/dre:552927/dre:564295/dre:140621/dre:58100/dre:559564/dre:561021/dre:246726/dre:558237 | 14 |
| dre00310 | Lysine degradation | 7/389 | 102/8379 | 0.1949198 | 0.7594997 | 100005587\_1/100536795/368262/393860\_1/556535\_1/100003615\_1/100006238\_1 | aldh9a1a.1/wu:fb14d09/dlst/gcdha/setd1a/prdm2a/aldh9a1a.2 | dre:100005587/dre:100536795/dre:368262/dre:393860/dre:556535/dre:100003615/dre:100006238 | 7 |
| dre00350 | Tyrosine metabolism | 4/389 | 50/8379 | 0.2011564 | 0.7594997 | 541429\_1/406688/393427/569363 | aoc2/got2a/zgc:63568/tpo | dre:541429/dre:406688/dre:393427/dre:569363 | 4 |
| dre00982 | Drug metabolism - cytochrome P450 | 6/389 | 85/8379 | 0.2017032 | 0.7594997 | 406731/100415796/570913\_1/406703/393427/767756 | ugt1ab/ugt5b5/ugt5g1/gsta.1/zgc:63568/ugt5a2 | dre:406731/dre:678597/dre:570913/dre:406703/dre:393427/dre:767756 | 6 |
| dre00620 | Pyruvate metabolism | 5/389 | 68/8379 | 0.2075052 | 0.7594997 | 100005587\_1/100536795/393427/445326/100006238\_1 | aldh9a1a.1/wu:fb14d09/zgc:63568/akr1a1a/aldh9a1a.2 | dre:100005587/dre:100536795/dre:393427/dre:445326/dre:100006238 | 5 |
| dre00830 | Retinol metabolism | 7/389 | 104/8379 | 0.2080821 | 0.7594997 | 406731/100415796/553239/570913\_1/393427/407663/767756 | ugt1ab/ugt5b5/lrata/ugt5g1/zgc:63568/si:ch211-107o10.3/ugt5a2 | dre:406731/dre:678597/dre:553239/dre:570913/dre:393427/dre:407663/dre:767756 | 7 |
| dre04370 | VEGF signaling pathway | 7/389 | 104/8379 | 0.2080821 | 0.7594997 | 793158/565241\_2/100000252\_1/58100\_1/100148818/497384\_1/792699 | cdc42l/ppp3ccb/pik3r3b/badb/si:ch73-55i23.1/prkca/rac1l | dre:793158/dre:565241/dre:100000252/dre:58100/dre:100148818/dre:497384/dre:792699 | 7 |
| dre04310 | Wnt signaling pathway | 16/389 | 278/8379 | 0.2205599 | 0.7731463 | 436718\_1/565241\_2/678649/323883/556087\_1/58072/30369\_1/791993/402843\_1/30127\_1/30197/100006858\_1/798564/326875\_1/497384\_1/792699 | rhocb/ppp3ccb/zgc:136929/cul1a/wnt2bb/fzd8b/fzd7a/wnt4b/sfrp1a/wnt2/dkk1b/csnk1e/sfrp1b/cby1/prkca/rac1l | dre:436718/dre:565241/dre:678649/dre:323883/dre:556087/dre:58072/dre:30369/dre:791993/dre:402843/dre:30127/dre:30197/dre:100006858/dre:798564/dre:326875/dre:497384/dre:792699 | 16 |
| dre05168 | Herpes simplex virus 1 infection | 13/389 | 220/8379 | 0.2224119 | 0.7731463 | 140621/373107\_1/100000252\_1/565338\_1/30400/564295\_2/140621\_1/406288\_1/58100\_1/559564/368481/30163/561021\_1 | casp3a/birc2/pik3r3b/eif2ak1/b2m/traf2a/casp3a/srsf1a/badb/eif2ak3/stat1b/tapbp.1/si:dkey-103e21.5 | dre:140621/dre:373107/dre:100000252/dre:565338/dre:30400/dre:564295/dre:140621/dre:406288/dre:58100/dre:559564/dre:368481/dre:30163/dre:561021 | 13 |
| dre03018 | RNA degradation | 7/389 | 108/8379 | 0.2353548 | 0.7991117 | 568001\_2/553665/324574/448949/566017/565659/561416 | pfkmb/cnot6l/wu:fc33b09/cnot1/lsm8/edc4/pfkpb | dre:568001/dre:553665/dre:324574/dre:448949/dre:566017/dre:565659/dre:561416 | 7 |
| dre04672 | Intestinal immune network for IgA production | 3/389 | 37/8379 | 0.2454158 | 0.8075692 | 799354\_1/100006078\_1/797491\_1 | cxcl12b/ccr9b/itgb7 | dre:799354/dre:100006078/dre:797491 | 3 |
| dre04216 | Ferroptosis | 5/389 | 73/8379 | 0.2504791 | 0.8075692 | 559768/558816/100126128/790938/100320152 | zgc:173593/LOC558816/zgc:173594/zgc:158423/steap3 | dre:559768/dre:558816/dre:100126128/dre:790938/dre:100320152 | 5 |
| dre05132 | Salmonella infection | 22/389 | 406/8379 | 0.2544396 | 0.8075692 | 386590/57934/30429\_2/793158/140621/436718\_1/373107\_1/406811\_1/798985/563023/436734\_1/562372/564295\_2/140621\_1/571356\_1/novel.848/100002946/445305\_1/561021\_1/403131/792699/799806\_2 | hsp90b1/actb1/mylpfa/cdc42l/casp3a/rhocb/birc2/zgc:55461/vps16/flnb/txn/snx18a/traf2a/casp3a/pik3c2a/-/cxcl8a/arpc4l/si:dkey-103e21.5/tlr4ba/rac1l/anxa2b | dre:386590/dre:57934/dre:30429/dre:793158/dre:140621/dre:436718/dre:373107/dre:336681/dre:798985/dre:563023/dre:436734/dre:562372/dre:564295/dre:140621/dre:571356/dre:393993/dre:100002946/dre:445305/dre:561021/dre:403131/dre:792699/dre:799806 | 22 |
| dre00360 | Phenylalanine metabolism | 2/389 | 22/8379 | 0.2722081 | 0.8160763 | 541429\_1/406688 | aoc2/got2a | dre:541429/dre:406688 | 2 |
| dre00450 | Selenocompound metabolism | 2/389 | 22/8379 | 0.2722081 | 0.8160763 | 777719/571201 | papss2a/si:ch73-97h19.2 | dre:777719/dre:571201 | 2 |
| dre04260 | Cardiac muscle contraction | 10/389 | 173/8379 | 0.2830292 | 0.8160763 | 393793\_1/64614/447942\_1/100037358\_1/559015/678560\_1/353247/556638\_1/114457/436768 | uqcrc1/atp1a1a.3/cox6a2/zgc:163073/cacnb3b/cacnb4b/tnnc1a/slc9a6b/atp1b2b/uqcrq | dre:393793/dre:64614/dre:447942/dre:100037358/dre:559015/dre:678560/dre:353247/dre:556638/dre:114457/dre:436768 | 10 |
| dre04510 | Focal adhesion | 19/389 | 354/8379 | 0.2882286 | 0.8160763 | 57934/30429\_2/793158/436718\_1/373107\_1/100000252\_1/386856\_1/563023/novel.946/556842\_1/571431/560564\_2/245702\_1/58100\_1/378480/797491\_1/796537\_1/497384\_1/792699 | actb1/mylpfa/cdc42l/rhocb/birc2/pik3r3b/pdgfra/flnb/-/tln2a/LOC571431/pdgfc/igf1rb/badb/pdgfaa/itgb7/kdrl/prkca/rac1l | dre:57934/dre:30429/dre:793158/dre:436718/dre:373107/dre:100000252/dre:386856/dre:563023/dre:386856/dre:556842/dre:571431/dre:560564/dre:245702/dre:58100/dre:103909601/dre:797491/dre:796537/dre:497384/dre:792699 | 19 |
| dre01240 | Biosynthesis of cofactors | 13/389 | 234/8379 | 0.2916401 | 0.8160763 | 100536795/403070/406731/100004977\_2/100415796/100007686/570913\_1/407663/64607\_1/334195/445326/767756/678634 | wu:fb14d09/rgn/ugt1ab/mthfd2/ugt5b5/LOC100007686/ugt5g1/si:ch211-107o10.3/alas2/mat1a/akr1a1a/ugt5a2/mat2al | dre:100536795/dre:403070/dre:406731/dre:100004977/dre:678597/dre:100007686/dre:570913/dre:407663/dre:64607/dre:334195/dre:445326/dre:767756/dre:678634 | 13 |
| dre04070 | Phosphatidylinositol signaling system | 9/389 | 157/8379 | 0.3059348 | 0.8160763 | 724010/368717\_1/100000252\_1/337236\_1/566642/559938/571356\_1/559198\_1/497384\_1 | dgkaa/itpka/pik3r3b/synj1/tmem55a/ppip5k1b/pik3c2a/mtmr7b/prkca | dre:724010/dre:368717/dre:100000252/dre:337236/dre:566642/dre:559938/dre:571356/dre:559198/dre:497384 | 9 |
| dre04530 | Tight junction | 16/389 | 300/8379 | 0.3188732 | 0.8160763 | 337226/57934/286739\_1/569084\_1/793158/436718\_1/81585/792828/60635\_1/100536545/110438642/556298/797651/445305\_1/559392/792699 | zgc:153867/actb1/msna/cldni/cdc42l/rhocb/cldnf/pard6a/cldn7b/synpo/LOC110438642/micall2b/jam3a/arpc4l/ppp2r2cb/rac1l | dre:337226/dre:57934/dre:286739/dre:569084/dre:793158/dre:436718/dre:81585/dre:792828/dre:60635/dre:100536545/dre:110438642/dre:556298/dre:797651/dre:445305/dre:559392/dre:792699 | 16 |
| dre00512 | Mucin type O-glycan biosynthesis | 3/389 | 43/8379 | 0.3222107 | 0.8160763 | 324510/557675/568500 | gcnt4a/c1galt1a/galnt8a.2 | dre:324510/dre:557675/dre:568500 | 3 |
| dre00330 | Arginine and proline metabolism | 5/389 | 81/8379 | 0.3230488 | 0.8160763 | 100005587\_1/100536795/321892/406688/100006238\_1 | aldh9a1a.1/wu:fb14d09/ckmt1/got2a/aldh9a1a.2 | dre:100005587/dre:100536795/dre:321892/dre:406688/dre:100006238 | 5 |
| dre00670 | One carbon pool by folate | 2/389 | 25/8379 | 0.3245110 | 0.8160763 | 100526683\_1/100004977\_2 | aldh1l1/mthfd2 | dre:100526683/dre:100004977 | 2 |
| dre03060 | Protein export | 2/389 | 25/8379 | 0.3245110 | 0.8160763 | 445299/436794\_2 | immp2l/sec11a | dre:445299/dre:436794 | 2 |
| dre04540 | Gap junction | 9/389 | 160/8379 | 0.3254367 | 0.8160763 | 386856\_1/406811\_1/novel.946/571431/560564\_2/378480/100000981\_2/566430\_1/497384\_1 | pdgfra/zgc:55461/-/LOC571431/pdgfc/pdgfaa/htr2cl1/prkg1b/prkca | dre:386856/dre:336681/dre:386856/dre:571431/dre:560564/dre:103909601/dre:100000981/dre:566430/dre:497384 | 9 |
| dre00500 | Starch and sucrose metabolism | 3/389 | 44/8379 | 0.3351114 | 0.8160763 | 406339\_1/100333665\_1/561803 | hk2/gbe1a/gyg2 | dre:406339/dre:100333665/dre:561803 | 3 |
| dre03460 | Fanconi anemia pathway | 4/389 | 63/8379 | 0.3353738 | 0.8160763 | 558923\_1/794038\_1/402936/100538153\_1 | palb2/brip1/fancg/slx4 | dre:558923/dre:794038/dre:402936/dre:100538153 | 4 |
| dre00240 | Pyrimidine metabolism | 5/389 | 84/8379 | 0.3509280 | 0.8362888 | 492517\_1/100005763\_1/437016\_1/103910413/393744\_2 | zgc:103586/uck2a/tk2/tymp/dctpp1 | dre:492517/dre:100005763/dre:437016/dre:103910413/dre:393744 | 5 |
| dre00630 | Glyoxylate and dicarboxylate metabolism | 3/389 | 46/8379 | 0.3608643 | 0.8362888 | 322670\_1/541329\_1/792824\_1 | aco2/gcsha/pgp | dre:322670/dre:541329/dre:792824 | 3 |
| dre03440 | Homologous recombination | 3/389 | 46/8379 | 0.3608643 | 0.8362888 | 558923\_1/794038\_1/554171 | palb2/brip1/rad52 | dre:558923/dre:794038/dre:554171 | 3 |
| dre00020 | Citrate cycle (TCA cycle) | 3/389 | 47/8379 | 0.3736900 | 0.8432980 | 322670\_1/550579/368262 | aco2/idh3g/dlst | dre:322670/dre:550579/dre:368262 | 3 |
| dre00400 | Phenylalanine, tyrosine and tryptophan biosynthesis | 1/389 | 10/8379 | 0.3785127 | 0.8432980 | 406688 | got2a | dre:406688 | 1 |
| dre04144 | Endocytosis | 21/389 | 417/8379 | 0.3812169 | 0.8432980 | 474327\_1/336426\_1/793158/436718\_1/792229/406684/792828/100003902\_1/550542\_1/406837/386856\_1/393881\_1/387529/560210/novel.946/571431/245702\_1/568170\_2/100151129/492487/445305\_1 | capza1b/chmp1b/cdc42l/rhocb/sh3glb1a/vps26b/pard6a/si:dkey-16l2.16/chmp4c/rnf41/pdgfra/vps26a/ldlra/hsp70l/-/LOC571431/igf1rb/eps15l1a/rab11fip4b/rab11a/arpc4l | dre:474327/dre:336426/dre:793158/dre:436718/dre:792229/dre:406684/dre:792828/dre:100003902/dre:550542/dre:406837/dre:386856/dre:393881/dre:387529/dre:30671/dre:386856/dre:571431/dre:245702/dre:568170/dre:100151129/dre:492487/dre:445305 | 21 |
| dre00561 | Glycerolipid metabolism | 5/389 | 89/8379 | 0.3975817 | 0.8501423 | 724010/100005587\_1/100536795/445326/100006238\_1 | dgkaa/aldh9a1a.1/wu:fb14d09/akr1a1a/aldh9a1a.2 | dre:724010/dre:100005587/dre:100536795/dre:445326/dre:100006238 | 5 |
| dre00600 | Sphingolipid metabolism | 5/389 | 89/8379 | 0.3975817 | 0.8501423 | 445250/445250\_1/100330155/100000684\_1/553981\_1 | neu3.2/neu3.2/cers4b/neu3.5/sptlc1 | dre:445250/dre:445250/dre:100330155/dre:100000684/dre:553981 | 5 |
| dre00740 | Riboflavin metabolism | 1/389 | 11/8379 | 0.4074001 | 0.8501423 | 436725 | acp5b | dre:436725 | 1 |
| dre03430 | Mismatch repair | 2/389 | 30/8379 | 0.4091025 | 0.8501423 | 568323/556995\_2 | mlh3/lig1 | dre:568323/dre:556995 | 2 |
| dre00140 | Steroid hormone biosynthesis | 5/389 | 91/8379 | 0.4161851 | 0.8501423 | 406731/393293/100415796/570913\_1/767756 | ugt1ab/hsd11b1la/ugt5b5/ugt5g1/ugt5a2 | dre:406731/dre:100006793/dre:678597/dre:570913/dre:767756 | 5 |
| dre00120 | Primary bile acid biosynthesis | 2/389 | 31/8379 | 0.4254270 | 0.8501423 | 723999/795106 | zgc:136333/cyp27a1.2 | dre:795106/dre:795106 | 2 |
| dre04623 | Cytosolic DNA-sensing pathway | 4/389 | 72/8379 | 0.4311255 | 0.8501423 | 140621/394162/140621\_1/561021\_1 | casp3a/polr3f/casp3a/si:dkey-103e21.5 | dre:140621/dre:394162/dre:140621/dre:561021 | 4 |
| dre04020 | Calcium signaling pathway | 22/389 | 450/8379 | 0.4322600 | 0.8501423 | 565241\_2/368717\_1/544664/386856\_1/566671/100005049/novel.946/569566/571431/560564\_2/387296\_1/58147\_1/378480/492486\_1/100000981\_2/353247/259258/796537\_1/100005590\_1/497384\_1/100137117/570493\_1 | ppp3ccb/itpka/erbb3b/pdgfra/orai1a/fgf1b/-/LOC569566/LOC571431/pdgfc/p2rx1/p2rx3a/pdgfaa/adra1bb/htr2cl1/tnnc1a/p2rx4a/kdrl/hrh2b/prkca/si:dkey-1h24.2/grin2ab | dre:565241/dre:368717/dre:544664/dre:386856/dre:566671/dre:100005049/dre:386856/dre:569566/dre:571431/dre:560564/dre:387296/dre:58147/dre:103909601/dre:492486/dre:100000981/dre:353247/dre:259258/dre:796537/dre:100005590/dre:497384/dre:100137117/dre:570493 | 22 |
| dre04146 | Peroxisome | 6/389 | 114/8379 | 0.4367170 | 0.8501423 | 450048/406803/562105/100093708/100003383\_1/110440136 | ech1/abcd3a/si:dkey-275b16.2/slc25a17/pmvk/LOC110440136 | dre:450048/dre:406803/dre:110440136/dre:100093708/dre:100003383/dre:110440136 | 6 |
| dre04137 | Mitophagy - animal | 6/389 | 116/8379 | 0.4533283 | 0.8708675 | 550134/100000242/555713\_1/336159\_1/559564/561933 | ubb/zgc:92606/nbr1b/optn/eif2ak3/rhot1b | dre:550134/dre:100000242/dre:555713/dre:336159/dre:559564/dre:561933 | 6 |
| dre03010 | Ribosome | 8/389 | 161/8379 | 0.4738971 | 0.8985582 | 550564\_1/768187\_1/553284/336712/554098\_2/450009\_1/553799/337007 | faub/mrpl18/mrpl1/rpl10/mrpl28/mrps16/mrpl30/rps12 | dre:550564/dre:768187/dre:553284/dre:336712/dre:554098/dre:450009/dre:553799/dre:337007 | 8 |
| dre04620 | Toll-like receptor signaling pathway | 6/389 | 121/8379 | 0.4943111 | 0.9078565 | 100000252\_1/368481/100002946/561021\_1/403131/792699 | pik3r3b/stat1b/cxcl8a/si:dkey-103e21.5/tlr4ba/rac1l | dre:100000252/dre:368481/dre:100002946/dre:561021/dre:403131/dre:792699 | 6 |
| dre03040 | Spliceosome | 8/389 | 164/8379 | 0.4949154 | 0.9078565 | 100004402/100005893\_1/566017/100037380\_2/560210/394058\_1/406288\_1/286774\_1 | plrg1/magoh/lsm8/zgc:163098/hsp70l/fus/srsf1a/phf5a | dre:100004402/dre:100005893/dre:566017/dre:100037380/dre:30671/dre:394058/dre:406288/dre:286774 | 8 |
| dre00250 | Alanine, aspartate and glutamate metabolism | 3/389 | 57/8379 | 0.4974556 | 0.9078565 | 447815\_1/564754\_1/406688 | rimkla/nat8l/got2a | dre:447815/dre:564754/dre:406688 | 3 |
| dre00562 | Inositol phosphate metabolism | 6/389 | 123/8379 | 0.5104242 | 0.9112793 | 560753/368717\_1/337236\_1/566479\_1/571356\_1/559198\_1 | tpi1b/itpka/synj1/plch1/pik3c2a/mtmr7b | dre:560753/dre:368717/dre:337236/dre:566479/dre:571356/dre:559198 | 6 |
| dre04261 | Adrenergic signaling in cardiomyocytes | 14/389 | 301/8379 | 0.5371221 | 0.9112793 | 64614/100333318/100037358\_1/793666/559015/678560\_1/562282\_1/555423/492486\_1/353247/550357\_2/114457/559392/497384\_1 | atp1a1a.3/creb3l4/zgc:163073/popdc2/cacnb3b/cacnb4b/ppp2r5b/scn1ba/adra1bb/tnnc1a/cremb/atp1b2b/ppp2r2cb/prkca | dre:64614/dre:100333318/dre:100037358/dre:793666/dre:559015/dre:678560/dre:562282/dre:555423/dre:492486/dre:353247/dre:550357/dre:114457/dre:559392/dre:497384 | 14 |
| dre03250 | Viral life cycle - HIV-1 | 4/389 | 83/8379 | 0.5423039 | 0.9112793 | 559677\_1/321602\_1/562808\_1/559032\_1 | nelfa/cdk9/map1sa/map1b | dre:559677/dre:321602/dre:562808/dre:559032 | 4 |
| dre04916 | Melanogenesis | 8/389 | 171/8379 | 0.5429342 | 0.9112793 | 100333318/556087\_1/58072/30369\_1/791993/393760\_1/30127\_1/497384\_1 | creb3l4/wnt2bb/fzd8b/fzd7a/wnt4b/gnao1a/wnt2/prkca | dre:100333318/dre:556087/dre:58072/dre:30369/dre:791993/dre:393760/dre:30127/dre:497384 | 8 |
| dre00515 | Mannose type O-glycan biosynthesis | 2/389 | 39/8379 | 0.5465588 | 0.9112793 | 569769/100093713 | pomt1/zgc:165519 | dre:569769/dre:100093713 | 2 |
| dre01040 | Biosynthesis of unsaturated fatty acids | 2/389 | 39/8379 | 0.5465588 | 0.9112793 | 334217/317738\_1 | elovl7a/elovl6 | dre:334217/dre:317738 | 2 |
| dre03320 | PPAR signaling pathway | 5/389 | 106/8379 | 0.5504832 | 0.9112793 | 550134/30355/723999/794841\_1/795106 | ubb/apoa1a/zgc:136333/plin2/cyp27a1.2 | dre:550134/dre:30355/dre:795106/dre:794841/dre:795106 | 5 |
| dre03013 | Nucleocytoplasmic transport | 6/389 | 129/8379 | 0.5575345 | 0.9112793 | 393693/100005893\_1/407710\_1/324342/100004503\_1/393973\_1 | sap18/magoh/xpo7/xpo4/eef1a1b/rae1 | dre:393693/dre:100005893/dre:407710/dre:324342/dre:100004503/dre:393973 | 6 |
| dre01232 | Nucleotide metabolism | 6/389 | 131/8379 | 0.5727686 | 0.9112793 | 492517\_1/100005763\_1/437016\_1/103910413/100005067\_1/393744\_2 | zgc:103586/uck2a/tk2/tymp/dguok/dctpp1 | dre:492517/dre:100005763/dre:437016/dre:103910413/dre:100005067/dre:393744 | 6 |
| dre04136 | Autophagy - other | 2/389 | 41/8379 | 0.5739362 | 0.9112793 | 100000242/792689\_1 | zgc:92606/mlst8 | dre:100000242/dre:792689 | 2 |
| dre00514 | Other types of O-glycan biosynthesis | 3/389 | 65/8379 | 0.5870126 | 0.9112793 | 557675/569769/568500 | c1galt1a/pomt1/galnt8a.2 | dre:557675/dre:569769/dre:568500 | 3 |
| dre03020 | RNA polymerase | 2/389 | 42/8379 | 0.5871673 | 0.9112793 | 394162/325603 | polr3f/polr1b | dre:394162/dre:325603 | 2 |
| dre04625 | C-type lectin receptor signaling pathway | 7/389 | 156/8379 | 0.5916477 | 0.9112793 | 436718\_1/565241\_2/100000252\_1/795414\_2/571242\_2/368481/561021\_1 | rhocb/ppp3ccb/pik3r3b/relb/malt3/stat1b/si:dkey-103e21.5 | dre:436718/dre:565241/dre:100000252/dre:795414/dre:571242/dre:368481/dre:561021 | 7 |
| dre00604 | Glycosphingolipid biosynthesis - ganglio series | 1/389 | 19/8379 | 0.5951399 | 0.9112793 | 394230\_1 | st3gal5 | dre:394230 | 1 |
| dre00565 | Ether lipid metabolism | 3/389 | 67/8379 | 0.6077502 | 0.9112793 | 406370\_1/767688/100148818 | pla2g6/zgc:154040/si:ch73-55i23.1 | dre:406370/dre:767688/dre:100148818 | 3 |
| dre00603 | Glycosphingolipid biosynthesis - globo and isoglobo series | 1/389 | 20/8379 | 0.6139784 | 0.9112793 | 100093713 | zgc:165519 | dre:100093713 | 1 |
| dre03420 | Nucleotide excision repair | 3/389 | 68/8379 | 0.6178562 | 0.9112793 | 393774\_2/556995\_2/553405\_1 | pole3/lig1/pole | dre:393774/dre:556995/dre:553405 | 3 |
| dre03022 | Basal transcription factors | 2/389 | 45/8379 | 0.6250223 | 0.9112793 | 798997\_2/567899 | gtf2a2/gtf2e1 | dre:798997/dre:567899 | 2 |
| dre04330 | Notch signaling pathway | 4/389 | 93/8379 | 0.6329056 | 0.9112793 | 678649/393500/557340/140422\_1 | zgc:136929/zgc:66440/atxn1a/jag2b | dre:678649/dre:393500/dre:557340/dre:140422 | 4 |
| dre00062 | Fatty acid elongation | 2/389 | 46/8379 | 0.6370294 | 0.9112793 | 334217/317738\_1 | elovl7a/elovl6 | dre:334217/dre:317738 | 2 |
| dre00280 | Valine, leucine and isoleucine degradation | 3/389 | 71/8379 | 0.6471055 | 0.9112793 | 100005587\_1/100536795/100006238\_1 | aldh9a1a.1/wu:fb14d09/aldh9a1a.2 | dre:100005587/dre:100536795/dre:100006238 | 3 |
| dre00520 | Amino sugar and nucleotide sugar metabolism | 3/389 | 74/8379 | 0.6747351 | 0.9112793 | 406339\_1/100007686/563138 | hk2/LOC100007686/si:ch211-219a15.4 | dre:406339/dre:100007686/dre:563138 | 3 |
| dre00100 | Steroid biosynthesis | 1/389 | 24/8379 | 0.6809841 | 0.9112793 | 494054\_1 | tm7sf2 | dre:494054 | 1 |
| dre00910 | Nitrogen metabolism | 1/389 | 24/8379 | 0.6809841 | 0.9112793 | 100006448 | ca6 | dre:100006448 | 1 |
| dre04520 | Adherens junction | 8/389 | 194/8379 | 0.6852015 | 0.9112793 | 57934/30429\_2/793158/436718\_1/569566/571431/245702\_1/792699 | actb1/mylpfa/cdc42l/rhocb/LOC569566/LOC571431/igf1rb/rac1l | dre:57934/dre:30429/dre:793158/dre:436718/dre:569566/dre:571431/dre:245702/dre:792699 | 8 |
| dre04140 | Autophagy - animal | 10/389 | 241/8379 | 0.6884032 | 0.9112793 | 321622/100000242/792229/100000252\_1/573492/792689\_1/245702\_1/58100\_1/559564/563140\_1 | hmgb1a/zgc:92606/sh3glb1a/pik3r3b/rragca/mlst8/igf1rb/badb/eif2ak3/si:ch211-11n16.2 | dre:110438871/dre:100000242/dre:792229/dre:100000252/dre:573492/dre:792689/dre:245702/dre:58100/dre:559564/dre:563140 | 10 |
| dre04141 | Protein processing in endoplasmic reticulum | 10/389 | 241/8379 | 0.6884032 | 0.9112793 | 327197/386590/266757\_1/323883/565338\_1/560210/564295\_2/100005358/559564/393877 | vcp/hsp90b1/tram1/cul1a/eif2ak1/hsp70l/traf2a/si:ch211-274f20.2/eif2ak3/derl3 | dre:327197/dre:386590/dre:266757/dre:323883/dre:565338/dre:30671/dre:564295/dre:100005358/dre:559564/dre:393877 | 10 |
| dre04622 | RIG-I-like receptor signaling pathway | 3/389 | 76/8379 | 0.6922526 | 0.9112793 | 564295\_2/100002946/561021\_1 | traf2a/cxcl8a/si:dkey-103e21.5 | dre:564295/dre:100002946/dre:561021 | 3 |
| dre00970 | Aminoacyl-tRNA biosynthesis | 2/389 | 51/8379 | 0.6925677 | 0.9112793 | 324940\_1/394188 | aars/qars | dre:792787/dre:394188 | 2 |
| dre01250 | Biosynthesis of nucleotide sugars | 2/389 | 51/8379 | 0.6925677 | 0.9112793 | 406339\_1/100007686 | hk2/LOC100007686 | dre:406339/dre:100007686 | 2 |
| dre00563 | Glycosylphosphatidylinositol (GPI)-anchor biosynthesis | 1/389 | 25/8379 | 0.6958371 | 0.9112793 | 561471 | pigl | dre:561471 | 1 |
| dre04012 | ErbB signaling pathway | 5/389 | 127/8379 | 0.7090222 | 0.9112793 | 100000252\_1/544664/571431/58100\_1/497384\_1 | pik3r3b/erbb3b/LOC571431/badb/prkca | dre:100000252/dre:544664/dre:571431/dre:58100/dre:497384 | 5 |
| dre04340 | Hedgehog signaling pathway | 3/389 | 79/8379 | 0.7171840 | 0.9112793 | 323883/30269\_1/100006858\_1 | cul1a/shha/csnk1e | dre:323883/dre:30269/dre:100006858 | 3 |
| dre00531 | Glycosaminoglycan degradation | 1/389 | 27/8379 | 0.7235057 | 0.9112793 | 565246 | si:dkey-192p21.6 | dre:565246 | 1 |
| dre04270 | Vascular smooth muscle contraction | 9/389 | 226/8379 | 0.7292289 | 0.9112793 | 337226/436718\_1/406370\_1/436966/403114/492486\_1/566430\_1/100148818/497384\_1 | zgc:153867/rhocb/pla2g6/zgc:91818/adm2a/adra1bb/prkg1b/si:ch73-55i23.1/prkca | dre:337226/dre:436718/dre:406370/dre:436966/dre:403114/dre:492486/dre:566430/dre:100148818/dre:497384 | 9 |
| dre00061 | Fatty acid biosynthesis | 1/389 | 28/8379 | 0.7363836 | 0.9112793 | 559001\_1 | fasn | dre:559001 | 1 |
| dre00770 | Pantothenate and CoA biosynthesis | 1/389 | 28/8379 | 0.7363836 | 0.9112793 | 100536795 | wu:fb14d09 | dre:100536795 | 1 |
| dre04514 | Cell adhesion molecules | 8/389 | 204/8379 | 0.7376105 | 0.9112793 | 569084\_1/81585/60635\_1/557935/110438642/797651/101886406/797491\_1 | cldni/cldnf/cldn7b/zmp:0000001082/LOC110438642/jam3a/cd28/itgb7 | dre:569084/dre:81585/dre:60635/dre:557935/dre:110438642/dre:797651/dre:101886406/dre:797491 | 8 |
| dre04110 | Cell cycle | 9/389 | 229/8379 | 0.7433128 | 0.9112793 | 431763\_1/323299\_2/323883/novel.902/797406\_2/563708/324475\_1/562282\_1/393223\_1 | gadd45ab/atrx/cul1a/-/espl1/skp2/smc3/ppp2r5b/fzr1a | dre:431763/dre:323299/dre:323883/dre:323299/dre:797406/dre:563708/dre:324475/dre:562282/dre:393223 | 9 |
| dre04912 | GnRH signaling pathway | 6/389 | 158/8379 | 0.7489967 | 0.9112793 | 793158/566945/30498/571431/100148818/497384\_1 | cdc42l/mmp14b/egr1/LOC571431/si:ch73-55i23.1/prkca | dre:793158/dre:566945/dre:30498/dre:571431/dre:100148818/dre:497384 | 6 |
| dre04145 | Phagosome | 8/389 | 209/8379 | 0.7614605 | 0.9167375 | 57934/359840\_1/406811\_1/571584/100005358/403131/792699/100007982 | actb1/atp6v1b2/zgc:55461/marco/si:ch211-274f20.2/tlr4ba/rac1l/hbl1 | dre:57934/dre:359840/dre:336681/dre:571584/dre:100005358/dre:403131/dre:792699/dre:100007982 | 8 |
| dre04068 | FoxO signaling pathway | 8/389 | 210/8379 | 0.7660409 | 0.9167375 | 324140/100000242/431763\_1/100000252\_1/571431/245702\_1/563708/100006858\_1 | sgk1/zgc:92606/gadd45ab/pik3r3b/LOC571431/igf1rb/skp2/csnk1e | dre:324140/dre:100000242/dre:431763/dre:100000252/dre:571431/dre:245702/dre:563708/dre:100006858 | 8 |
| dre02010 | ABC transporters | 2/389 | 63/8379 | 0.7975882 | 0.9436281 | 406803/553281 | abcd3a/abcc8 | dre:406803/dre:553281 | 2 |
| dre01212 | Fatty acid metabolism | 3/389 | 91/8379 | 0.8014376 | 0.9436281 | 334217/559001\_1/317738\_1 | elovl7a/fasn/elovl6 | dre:334217/dre:559001/dre:317738 | 3 |
| dre00220 | Arginine biosynthesis | 1/389 | 35/8379 | 0.8112422 | 0.9475309 | 406688 | got2a | dre:406688 | 1 |
| dre04114 | Oocyte meiosis | 6/389 | 182/8379 | 0.8556657 | 0.9859121 | 565241\_2/323883/797406\_2/245702\_1/324475\_1/562282\_1 | ppp3ccb/cul1a/espl1/igf1rb/smc3/ppp2r5b | dre:565241/dre:323883/dre:797406/dre:245702/dre:324475/dre:562282 | 6 |
| dre04218 | Cellular senescence | 8/389 | 234/8379 | 0.8576084 | 0.9859121 | 565241\_2/431763\_1/100000252\_1/393176\_1/557551/100037341/100002946/572042 | ppp3ccb/gadd45ab/pik3r3b/rad9a/hipk2/zgc:162895/cxcl8a/hipk1a | dre:565241/dre:431763/dre:100000252/dre:393176/dre:557551/dre:100037341/dre:100002946/dre:572042 | 8 |
| dre00601 | Glycosphingolipid biosynthesis - lacto and neolacto series | 1/389 | 42/8379 | 0.8648822 | 0.9865062 | 100093713 | zgc:165519 | dre:100093713 | 1 |
| dre04142 | Lysosome | 7/389 | 214/8379 | 0.8749288 | 0.9873050 | 571066/406511/553423\_1/337771/436725/565246/445244\_1 | si:ch1073-513e17.1/sort1a/sumf1/zgc:162858/acp5b/si:dkey-192p21.6/ap1m3 | dre:571066/dre:406511/dre:553423/dre:337771/dre:436725/dre:565246/dre:445244 | 7 |
| dre00564 | Glycerophospholipid metabolism | 4/389 | 135/8379 | 0.8791072 | 0.9873050 | 724010/406370\_1/767688/100148818 | dgkaa/pla2g6/zgc:154040/si:ch73-55i23.1 | dre:724010/dre:406370/dre:767688/dre:100148818 | 4 |
| dre03008 | Ribosome biogenesis in eukaryotes | 2/389 | 83/8379 | 0.9036413 | 0.9908680 | 393107/407084 | utp6/riok2 | dre:393107/dre:407084 | 2 |
| dre00190 | Oxidative phosphorylation | 5/389 | 171/8379 | 0.9045810 | 0.9908680 | 393793\_1/359840\_1/447942\_1/796997\_1/436768 | uqcrc1/atp6v1b2/cox6a2/ndufs7/uqcrq | dre:393793/dre:359840/dre:447942/dre:796997/dre:436768 | 5 |
| dre04814 | Motor proteins | 10/389 | 305/8379 | 0.9075009 | 0.9908680 | 474327\_1/337226/57934/30429\_2/334274/100149148/406811\_1/100037358\_1/353247/556023 | capza1b/zgc:153867/actb1/mylpfa/myhc4/myha/zgc:55461/zgc:163073/tnnc1a/dnah2 | dre:474327/dre:337226/dre:57934/dre:30429/dre:334274/dre:100149148/dre:336681/dre:100037358/dre:353247/dre:556023 | 10 |
| dre00480 | Glutathione metabolism | 2/389 | 85/8379 | 0.9107540 | 0.9908680 | 553575\_1/406703 | gsr/gsta.1 | dre:553575/dre:406703 | 2 |
| dre04130 | SNARE interactions in vesicular transport | 1/389 | 56/8379 | 0.9308244 | 0.9908680 | 393294 | use1 | dre:393294 | 1 |
| dre04910 | Insulin signaling pathway | 6/389 | 212/8379 | 0.9337557 | 0.9908680 | 100001260/100000252\_1/79380\_1/406339\_1/58100\_1/559001\_1 | ppp1r3ab/pik3r3b/eif4ea/hk2/badb/fasn | dre:100001260/dre:100000252/dre:79380/dre:406339/dre:58100/dre:559001 | 6 |
| dre04744 | Phototransduction | 1/389 | 57/8379 | 0.9340575 | 0.9908680 | 100330921 | si:ch211-103a14.5 | dre:100330921 | 1 |
| dre04914 | Progesterone-mediated oocyte maturation | 3/389 | 133/8379 | 0.9504862 | 0.9908680 | 100000252\_1/245702\_1/393223\_1 | pik3r3b/igf1rb/fzr1a | dre:100000252/dre:245702/dre:393223 | 3 |
| dre03050 | Proteasome | 1/389 | 65/8379 | 0.9550456 | 0.9908680 | 796450 | adrm1 | dre:796450 | 1 |
| dre04371 | Apelin signaling pathway | 6/389 | 226/8379 | 0.9552197 | 0.9908680 | 30498/100331581\_1/335655/100037358\_1/321449\_1/557486 | egr1/si:dkey-44g17.6/gngt2a/zgc:163073/ctgfa/hdac5 | dre:30498/dre:100331581/dre:335655/dre:100037358/dre:321449/dre:557486 | 6 |
| dre00510 | N-Glycan biosynthesis | 1/389 | 68/8379 | 0.9610658 | 0.9908680 | 327601\_1 | alg8 | dre:327601 | 1 |
| dre04920 | Adipocytokine signaling pathway | 2/389 | 108/8379 | 0.9639462 | 0.9908680 | 100037357/564295\_2 | tnfrsf1b/traf2a | dre:100037357/dre:564295 | 2 |
| dre04060 | Cytokine-cytokine receptor interaction | 6/389 | 242/8379 | 0.9719189 | 0.9908680 | 100037357/799354\_1/368326/100006078\_1/562247\_1/100002946 | tnfrsf1b/cxcl12b/il13ra2/ccr9b/clcf1/cxcl8a | dre:100037357/dre:799354/dre:368326/dre:100006078/dre:562247/dre:100002946 | 6 |
| dre04350 | TGF-beta signaling pathway | 3/389 | 161/8379 | 0.9821030 | 0.9908680 | 436718\_1/323883/556634 | rhocb/cul1a/adamtsl7 | dre:436718/dre:323883/dre:556634 | 3 |
| dre04512 | ECM-receptor interaction | 2/389 | 128/8379 | 0.9840812 | 0.9908680 | 563428/797491\_1 | fras1/itgb7 | dre:563428/dre:797491 | 2 |
| dre00230 | Purine metabolism | 2/389 | 192/8379 | 0.9989686 | 0.9989686 | 777719/100005067\_1 | papss2a/dguok | dre:777719/dre:100005067 | 2 |
